# Bone marrow mesenchymal stem cell-derived exosomes shuttle microRNAs to endometrial stromal fibroblasts that promote tissue proliferation /regeneration/ and inhibit differentiation

**DOI:** 10.1186/s13287-024-03716-1

**Published:** 2024-05-01

**Authors:** Giulia Bonavina, Ramanaiah Mamillapalli, Graciela Krikun, Yuping Zhou, Nimisha Gawde, Hugh S. Taylor

**Affiliations:** 1 Department of Obstetrics, Gynecology and Reproductive Sciences, Yale School of Medicine, 310 Cedar Street, 06510 New Haven, CT USA; 2 Present Address: IRCCS San Raffaele Scientific Institute, Milan, Italy

**Keywords:** BMDSCs, eSF, Decidualization, miRNAs, Exosomes

## Abstract

**Background:**

Human bone marrow-derived stem cells (hBMDSCs) are well characterized mediators of tissue repair and regeneration. An increasing body of evidence indicates that these cells exert their therapeutic effects largely through their paracrine actions rather than clonal expansion and differentiation. Here we studied the role of microRNAs (miRNAs) present in extracellular vesicles (EVs) from hBMDSCs in tissue regeneration and cell differentiation targeting *endometrial stromal fibroblasts (eSF)*.

**Methods:**

Extracellular vesicles (EVs) are isolated from hBMDSCs, characterized by transmission electron microscopy (TEM) and nanoparticle tracking analysis (NTA) techniques. Extracted total RNA from EVs was subjected to RNA seq analysis. Transfection and decidualization studies were carried out in *endometrial stromal fibroblasts (eSF)*. Gene expression was analyzed by qRTPCR. Unpaired t-test with Welch’s correction was used for data analysis between two groups.

**Results:**

We identified several microRNAs (miRNAs) that were highly expressed, including miR-21-5p, miR-100-5p, miR-143-3p and let7. MiR-21 is associated with several signaling pathways involved in tissue regeneration, quiescence, cellular senescence, and fibrosis. Both miR-100-5p and miR-143-3p promoted cell proliferation. MiR-100-5p specifically promoted regenerative processes by upregulating *TGF-ß3*, *VEGFA*, *MMP7*, and *HGF*. MiR-100-5p blocked differentiation or decidualization as evidenced by morphologic changes and downregulation of decidualization mediators including *HOXA10*, *IGFBP*1, *PRL*, *PR-B*, and *PR*.

**Conclusion:**

EVs delivered to tissues by hBMDSCs contain specific miRNAs that prevent terminal differentiation and drive repair and regeneration. Delivery of microRNAs is a novel treatment paradigm with the potential to replace BMDSCs in cell-free regenerative therapies.

**Supplementary Information:**

The online version contains supplementary material available at 10.1186/s13287-024-03716-1.

## Background

Regenerative capacity of organs and tissue is an essential biological process for the long-term survival of living organisms [1, 2]. In adults, tissue regeneration after injury usually leads to scar formation and abnormal tissue re-organization due to loss of regenerative capacity during development and aging [3, 4]. Few organs can fully regenerate without scarring [5]. The extent of this regenerative capacity varies considerably among different tissues, which possess distinct and inherent cellular turnover potential [5]. However, adult tissue turnover is essential for the maintenance of multicellular living organisms in order to preserve and restore their morphology and function or to respond to external injury and stress. Adult mesenchymal stem cells (MSCs) are pluripotent undifferentiated cells responsible for homeostasis as well as regeneration of specific mammalian tissues [6]. MSCs have a unique capacity for either symmetric or asymmetric division that allows for both self-renewal and the generation of mature functional cells in a cell-lineage-specific fashion [6]. Surprisingly, adult stem cells are rare, often quiescent, proliferate less and function in a paracrine manner in various physiological processes [7].

Bone marrow derived mesenchymal stem cells (BMDSCs) are a source of adult multipotent stem cells that can differentiate and influence multiple tissues due to their ability to travel to distant sites in response to factors released from target tissues [8]. They also secrete bioactive molecules that promote tissue regeneration as well as stimulate proliferation or differentiation of endogenous progenitors. The paradigm has shifted toward a bidirectional secretome-based paracrine activity, rather than cellular engraftment and differentiation as the principal action of MSCs. MSCs shuttle extracellular vesicles (EVs) that underly some of their physiological and pathological effects [9]. EVs have been detected in almost all human cells (including stem cells [10] and in multiple biofluids [11] - and have a role in both normal and pathological conditions [12]. EVs, particularly exosomes, are a heterogeneous spherical lipid bilayer membrane nanoparticles that constitute a refined system of cell-to-cell communication characterized by transfer of biomolecules (proteins, lipids, nucleic acids and metabolites) from donor to recipient cell where they can influence cell fate, function, and plasticity in the target tissue [13–15]. Exosomes are secreted by most cells in the body and are formed through a series of steps such as invagination of the plasma membrane, budding of intracellular organelle membranes, exocytosis of intraluminal vesicles [16, 17] into the extracellular matrix, or circulation. The secreted or released exosomes reach their target cells through the circulation and are then internalized by the recipient cells [2]. Then, the components of exosomes are released into the recipient cell cytoplasm, which results in the activation of intracellular signaling pathways that modulate cellular processes and functions. Exosomes from hBMDSCs may contain specific biological molecules, including microRNAs (miRNAs), which exert specific effects on recipient cells that mediate the therapeutic effects of hBMDSCs [18].

The endometrium, the mucosal inner layer of the uterus, is unique in regenerating its tissue. Endometrial regeneration is a highly coordinated, carefully controlled, hormonal-dependent process during a woman’s reproductive life. Previously, we reported the first evidence of bone marrow’s contribution to regeneration of both stromal and epithelial cells in the endometrium [19], which was confirmed in multiple subsequent studies [20, 21]. While stromal decidual differentiation is crucial for pregnancy [22], but sloughed during the menstrual cycle, parturition, or injury [23–25], the endometrium regenerates repetitively [26]. Moreover, recent studies suggest that BMDSC engraftment dramatically increases during pregnancy, where they are crucial for successful uterine angiogenesis and stromal decidual differentiation [27, 28]. However, the role and mechanism by which BMDSC remodel tissues is still poorly understood. Here we investigated the role of miRNAs from EVs derived from hBMDSCs and identified their role in the regulation of tissue regeneration using endometrial differentiation and repair in an *in-vitro* model.

## Materials and methods

### Extracellular vesicles (EVs) isolation from h-BMDSCs

Human bone marrow derived mesenchymal stem cells (hBMDSCs) were obtained from ATCC (American Type Culture Collection, Manassas, Virginia, USA). The cells were grown in T75 tissue culture flasks with mesenchymal stem cell basal medium (ATCC) with 10% fetal bovine serum, and 1% antibiotic-antimycotic. Cells were allowed to reach 70–80% confluence for 48 hr and then cells were maintained in serum free media for 24 hr before supernatant was collected. EVs were collected from the supernatants of h-BMDSCs according to the MISEV2018 guidelines. EVs were isolated and purified from the supernatants collected, using ExoQuick-TC® ULTRA kit (Systems Biosciences, Palo Alto, CA, USA) according to the manufacturer protocol. We also used ultracentrifugation method for the isolation of EVs. Both methods provided a similar yield (Supplemental Fig. 1).

### Characterization of EVs

The nature of the isolated EVs was confirmed by Transmission Electron Microscopy (TEM). Briefly, EVs were mixed with 4% paraformaldehyde (Sigma Aldrich, St. Louis, MO, USA) for 30 min, counterstained with Alcian blue (Sigma Aldrich) for 30 min, washed with phosphate buffered saline (PBS) four times and centrifuged at 15,000 *g* for 5 min. EV pellets were fixed in 2% glutaraldehyde for 5 min. Resuspended EV mixtures were transferred to 300-mesh formvar nickel grids and incubated for 40 min. All grids were negatively stained with 4% saturated aqueous uranyl acetate for 15 min and then analyzed using an FEI Tecnai Osiris transmission electron microscope at 200 kV (FEI). All EVs detected were subjected to morphometric analysis and the exosome size and quantification were determined by nanoparticle tracking analysis (NTA) using ZetaView PMX 110 (Particle Metrix, Meerbusch, German). Data analysis was performed using ZetaView software (Particle Metrix, Meerbusch, German).

#### RNA-Seq

Total RNA was isolated from EVs by Qiagen RNeasy Plus Micro Kit, and RNA concentration was determined by NanoDrop 2000 (Thermo Fisher Scientific). RNA quality analysis, library preparation, and sequencing were performed by the Yale Center for Genomic Analysis. RNA was measured using an Agilent 2000 Bioanalyzer, which utilized the Agilent RNA 6000 Pico Chip (Agilent, Santa Clara, CA) per the manufacturer’s specifications. Illumina TrueSeq Library Preparation Kit (Illumina, San Diego, CA) was used for RNA-seq library preparation in accordance with the manufacturer’s protocol. Random primers were used for first-strand synthesis and then second-strand synthesis was performed with dUTP for generating strand-specific sequencing libraries. The parameters were set for single-end chemistry, high output, and 56-bp sequencing. Samples were multiplexed to six samples per lane. Libraries were sequenced on an Illumina HiSeq2500.

### Transfection of eSFs with miRNAs

Human endometrial stromal cells^23^ (eSFs) were cultured in Dulbecco Modified Eagle Medium (DMEM) with high glucose, 10% fetal bovine serum (FBS), and 1% antibiotic-antimycotic (Life Technologies, Carlsbad, CA, USA) at 37 °C and 5% CO_2_. Cells were seeded into a twelve-well plate (4.0 × 10^4^ cells/well). Transfection was carried out after 16 to 18 hr, at 30–40% confluence, with hsa-miR- 21-5p, hsa-miR-100-5p, hsa-miR-143-3p, hsa-let-7i-5p, hsa-let-7b-5p mimics or inhibitors (50 nM), or the respective negative controls (Bioneer Inc., Daejeon, South Korea) using the Lipofectamine RNAiMax transfection reagent (Invitrogen, Carlsbad, CA, USA) according to the manufacturer’s protocol. After 72 hr, the cells were harvested, and the number of live cells was counted using a hemocytometer with the trypan blue staining method. Seventy-two hr after transfection, total RNA was isolated from cells and cDNA was prepared.

### Induction of decidualization

To induce decidualization in vitro, both transfected and non-transfected eSFs were cultured in DMEM medium for 24 hr. Medium was replaced when cells reached to 70–80% confluence. The replaced medium containing 0.5 mM 8-Br-cAMP (Sigma-Aldrich) and 1 µM medroxyprogesterone acetate (MPA) (Sigma-Aldrich) was maintained for 7 days. The conditioned medium was collected and replaced every 24 hr and stored at -20 °C. Prolactin (PRL) protein levels were determined by using a commercially available ELISA kit (R&D Systems, Minneapolis, MN, US). eSFs were observed under an inverted microscope every 24 hr to study their morphological changes.

### Quantitative real-time polymerase chain reaction (qRT-PCR)

Total RNA was isolated from transfected and non-transfected eSFs using RNeasy Plus Micro kit (Qiagen, cat. #74 − 34, Germantown, MA, USA). RNA (400 ng) was reverse transcribed to cDNA in a 20 µL reaction mixture using the iScript cDNA Synthesis Kit (Bio-Rad Laboratories). The specific primers used for each gene are shown in Table 1. As PR-A is truncated by 164 amino acids in the *N*-terminal regulatory domain, primers can only be designed for PR and PR-B (relative PR-A expression is calculated by PR minus PR-B expression). Gene expression was carried out by qRT-PCR using SYBR Green real-time PCR Master Mix (Bio-Rad Laboratories) to measure the mRNA levels of Ki67, HOXA10, IGFBP-1, PRL, PGR, PGR-B, VEGFA, TGFβ3, MMP7, HGF, GREM2, LTBP1, VEGFC, SCLC43A2, and IL6R. SYBR Green (Bio-Rad) was optimized in the MyiQ Single Color Real-Time PCR Detection System (Bio-Rad). qRT-PCR was carried out for 39 cycles of denaturation at 95◦C for 10 s, following activation at 95◦C for 3 min, and annealing at 60 °C for 30 s. The specificity of the amplified transcript and absence of primer-dimers was confirmed by a melting curve analysis. Gene expression was normalized to GAPDH as an internal control. Relative mRNA expression was calculated using the comparative cycle threshold method (CT), also known as 2^−ΔΔCT^ method. Each sample was analyzed in triplicate, and all experiments were repeated at least three times. Nuclease-free water was used as a negative control replacing the cDNA template.

**Table 1:** Primer sequences used for qRTPCR

Gene	Forward	Reverse
Ki-67	5’ TTTGGGTGCGACTTGACGAG 3’	5’ CGTCCAGCATGTTCTGAGGA 3’
HOXA10	5’ AGGTGGACGCTGCGGCTAATCTCTA 3’	5’ GCCCCTTCCGAGAGCAGCAAAG 3’
IGFBP-1	5’ ATGGCTCGAAGGCTCTCCAT 3’	5’ TCCTGTGCCTTGGCTAAACTC 3’
PRL	5’ CATCAACAGCTGCCACACTT 3’	5’ CGTTTGGTTTGCTCCTCAAT 3’
PGR	5’ CAGCCAGAGCCCACAATACA 3’	5’ GCTCCCACAGGTAAGGACAC 3’
PGR-B	5’ GGAATGGGCTGTACCGAGAG 3’	5’ CGGCTCCTTTATCTCCCGAC 3’
VEGFA	5’ ATGCGGATCAAACCTCACCA 3’	5’ CACCAACGTACACGCTCCAG 3’
TGFβ3	5’ GTGCCGTGAACTGGCTTCT 3’	5’ CCAGTGAGTAGGTGGGGAGA 3’
MMP7	5’ TGGACGGATGGTAGCAGT 3’	5’ CAGAGGAATGTCCCATACC 3’
HGF	5’ TGATACCACACGAACACAGC 3’	5’ AGGCTGGGTACAAAGACAGC 3’
GAPDH	5’ TCAAGAAGGTGGTGSSGCAGG 3’	5’ TCAAAGGTGGAGGAGTGGGT 3’
GREM2	5’ AAGGCAGAGGGAGAGGGAGA3’	5’ CACCAGGAACAAGGACAGGGA 3’
LTBP1	5’ AATGGTCATGCTGCCGACAC 3’	5’ CTGCACTGGCCACCATTCATAC 3’
VEGFC	5’ CAGTTACGGTCTGTGTCCAGTGTAG 3’	5’ GGACACACATGGAGGTTTAAAGAAGC 3’
SLC43A2	5’ TTATCGCCTTGGCTCTGAAC 3’	5’ GGAAGCGTAGGATCCAATCA 3’
IL6R	5’ CCCCTCAGCAATGTTGTTTGT 3’	5’ CTCCGGGACTGCTAACTGGT 3’

### Statistical analysis

GraphPad Prism version 9.4.1 (458) (San Diego, CA, USA) was used for statistical analysis. Student t-test was used to compare between two groups after determining homogeneity of variance. Data were represented as mean ± standard error of the mean (SEM). *p* < 0.05 was considered statistically significant.

## Results

### Characterization of EVs from hBMDSCs

**Fig. 1:**
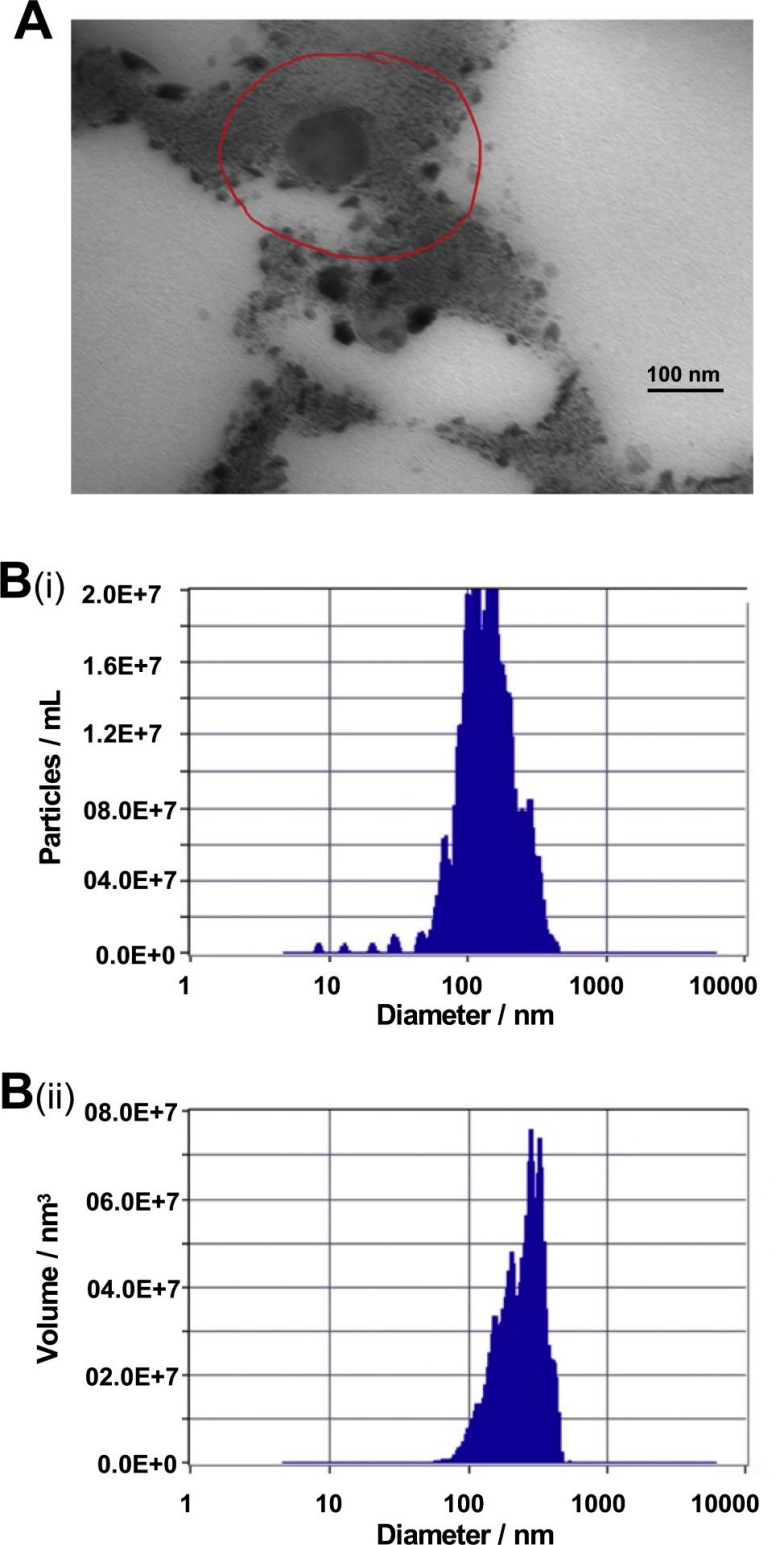
Characterization of extracellular vesicles (EVs). (**a**) Morphology of EVs showing a typical cup-shaped structure with a bilayer membrane and a diameter of approximately 100 nm, measured by transmission electron microscopy (TEM). (**b** i & ii) Shows the main peak and the median particle size respectively, in the range of exosomes determined by the nanoparticle tracking analysis (NTA)

EVs isolated and purified from the supernatants of hBMDSCs were characterized by transmission electron microscopy (TEM). TEM showed a typical cup-shaped morphology with a bilayer membrane and a diameter of approximately 100 nm (Fig. 1a). Additionally, nanoparticle tracking analysis (NTA) showed that the main peak and the median particle size was in the range of exosomes as shown in Fig. 1b **(**i) and (ii) respectively.

### MiRNAs in exosomes from hBMDSCs

**Fig. 2:**
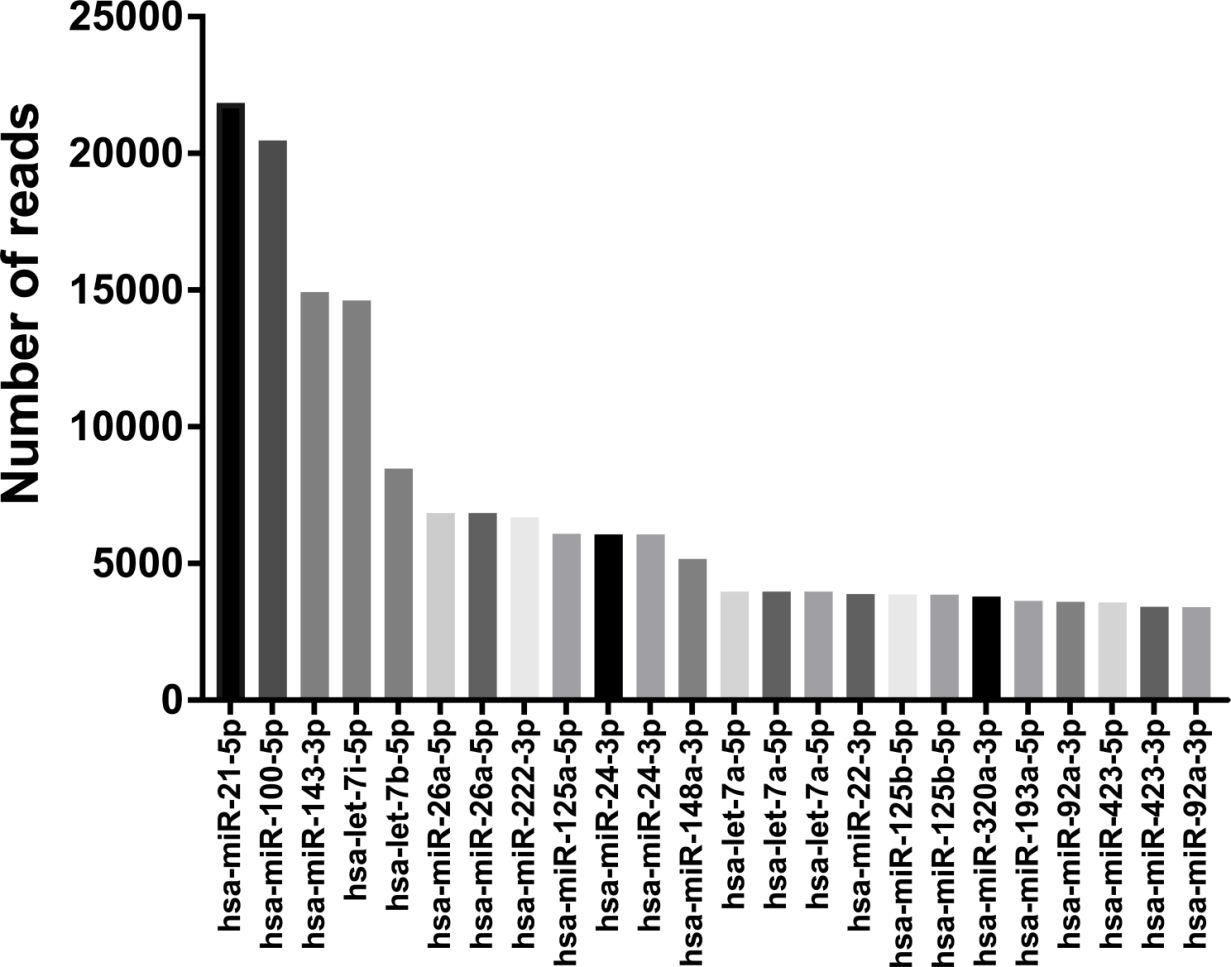
Analysis of RNA-seq. RNA seq analysis showing the top 25 miRNAs that are highly expressed in extracellular vesicles from BMDSCs. *MiR-21-5p* is most highly expressed followed by *miR-100-5p*, *miR-143-3p*, *miR-let-7i*, and *miR-let-7b*, with a minimum of 5000 copies

In order to know the presence and expression pattern of miRNAs in exosomes derived from hBMDSCs, we extracted exosomes from hBMDSCs using both ultracentrifugation and a commercial isolation kit utilizing RNA-seq focused specifically on small RNAs. Both methods showed similar pattern of miRNAs (Supplementary Fig. 1). RNA-seq data analysis identified a total of 2888 miRNAs. Of those 409 miRNAs were consistently expressed in multiple experiments. Among 409 miRNAs, the 25 most abundant are shown in Fig. 2. MiR-21-5p was found to be most abundant followed by miR-100-5p, miR-143-3p, miR-let-7i and miR-let-7b. We studied the first three miRs (miR-21, miR-100 and miR-143) for their role in tissue regeneration and differentiation.

### Effects of hsa-miR-21-5p on gene expression

RNA-seq data showed miR-21-5p to be the most abundant miRNA in hBMDSCs-derived EVs. To assess the effects of this miRNA on gene expression in tissue reprogramming, we transfected either miR-21-5p or its mimic negative control in eSFs. RNA sequencing was used to identify differentially expressed genes by using cut-offs of fold change > 1.2 or ≤ 0.83; P value < 0.05. We found that 66 genes are differentially expressed in eSFs transfected with miR-21-5p compared to the mimic negative control. Among these, 32 genes are upregulated while 34 are downregulated (Table 2). To determine the role of these genes in various physiological processes, we carried out Ingenuity Pathway Analyses (IPA). IPA results showed 14 genes to have roles in regeneration, cell cycle quiescence, cell differentiation, migration and/or angiogenesis. Among these 14 genes, 8 upregulated (*CEMIP, BDKRB1, DDIT4, GREM2, ADM2, KRTAP1-5, VEGFC, and SCUBE3*) and 6 downregulated (*FAM47E-STBD1, NAP1L5, PPP1R3B, PLK2, ERRFI1* and *CDC25A*). We also analyzed these genes for their involvement in specific signaling pathways (Supplementary Fig. 2) that are involved in tissue regeneration, cellular senescence reversal, fibrosis attenuation, and stress response. Among the pathways identified by IPA analysis, the IL-17 signaling pathway is one that regulates inflammation, metabolism, fibrosis, and aging. The genes that are involved in the IL-17 signaling pathway were analyzed by qRT-PCR. Among them, GREM2 (*p* = 0.0009), LTBP1 (*p* = 0.048), VEGFC (*p* = 0.044) and SLC43A2 (*p* = 0.017) were significantly upregulated while IL6R (*p* < 0.0001), was downregulated significantly as shown in supplementary Fig. 3.

**Fig. 3:**
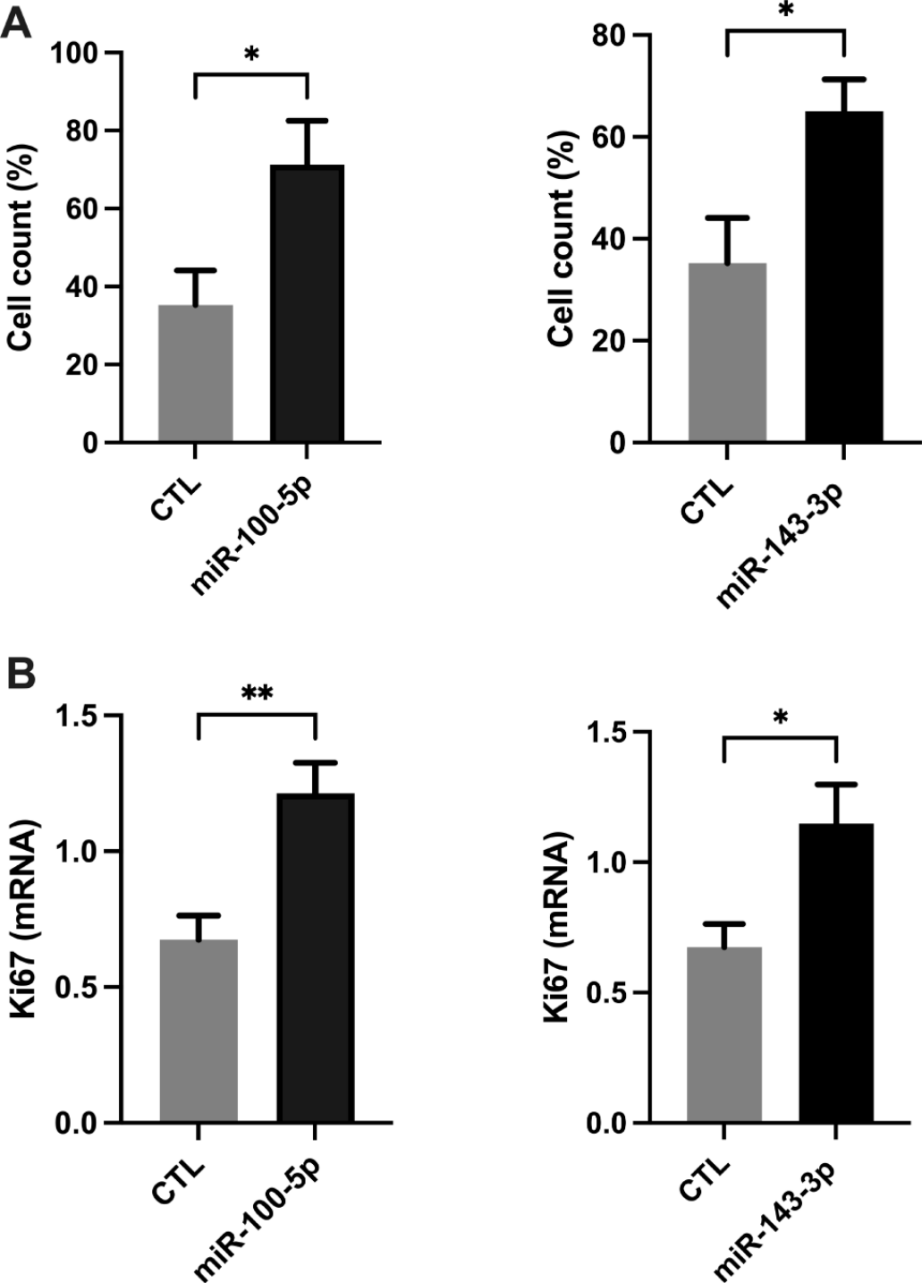
Increased cell proliferation by hsa-miR-100-5p and miR-143-3p. (**a**) Shows the significant increase in cell counts and (**b**) shows significant increase in Ki67 mRNA levels compared to mimic-negative control (CTL) for 72 hr. Each bar represents the data from six individual experiments, and each experiment was performed in duplicate. Data are shown as mean ± standard error (SEM) CTL vs. mimic (** *p* < 0.01, *p < 0.05)

**Table 2:** Up- and down regulated genes in eSFs transfected with miR-21

Upregulated genes	Downregulated genes
INO80B-WBP1	AC005324.3
AC120057.3	ISY1-RAB43
AC026362.1	FAM47E-STBD1
BEST1	RPS10-NUDT3
AC139530.2	IL6R
HYPK	SCHIP1
CEMIP	SKP2
GAPLINC	NAP1L5
BDKRB1	NCR3LG1
DDIT4	PPP1R3B
AL136295.5	ANKRD1
GREM2	DDAH1
SLC16A6	TGFB2
ADM2	AC010547.5
SLC6A9	AL136295.4
AP002990.1	ARNTL
KCNC4	TDG
CHAC1	AC117402.1
PTGES3L-AARSD1	CD68
KRTAP1-5	AC093668.2
STBD1	GCNT2
AL359736.1	KBTBD8
UCP2	GBP3
MRPS14	PLK2
VEGFC	PPARA
SCUBE3	ALDH1A1
CCL2	PALM2-AKAP2
SLC43A2	TUFT1
LTBP1	COBLL1
AC134772.2	ERRFI1
MLEC	RP2
PCK2	PAG1
	CDC25A
	AL807752.7

### MiR-100-5p and miR-143-3p promoted endometrial proliferation

We tested the effect of miR-100-5p, miR-143-3p, miR-let-7i-5p, and let-7b-5p on cell proliferation and differentiation. Figure 3a & b showed a significant increase (*p* < 0.05) in cell proliferation by miR-100-5p and miR-143-3p as determined by cell count (2-fold). Additionally, qRT-PCR showed an increase in Ki67 mRNA expression levels (1.75-fold). Transfection of miR-21-5p, let-7i-5p and miR-let-7b-5p did not show any significant effect on cell proliferation of eSFs.

**Fig. 4:**
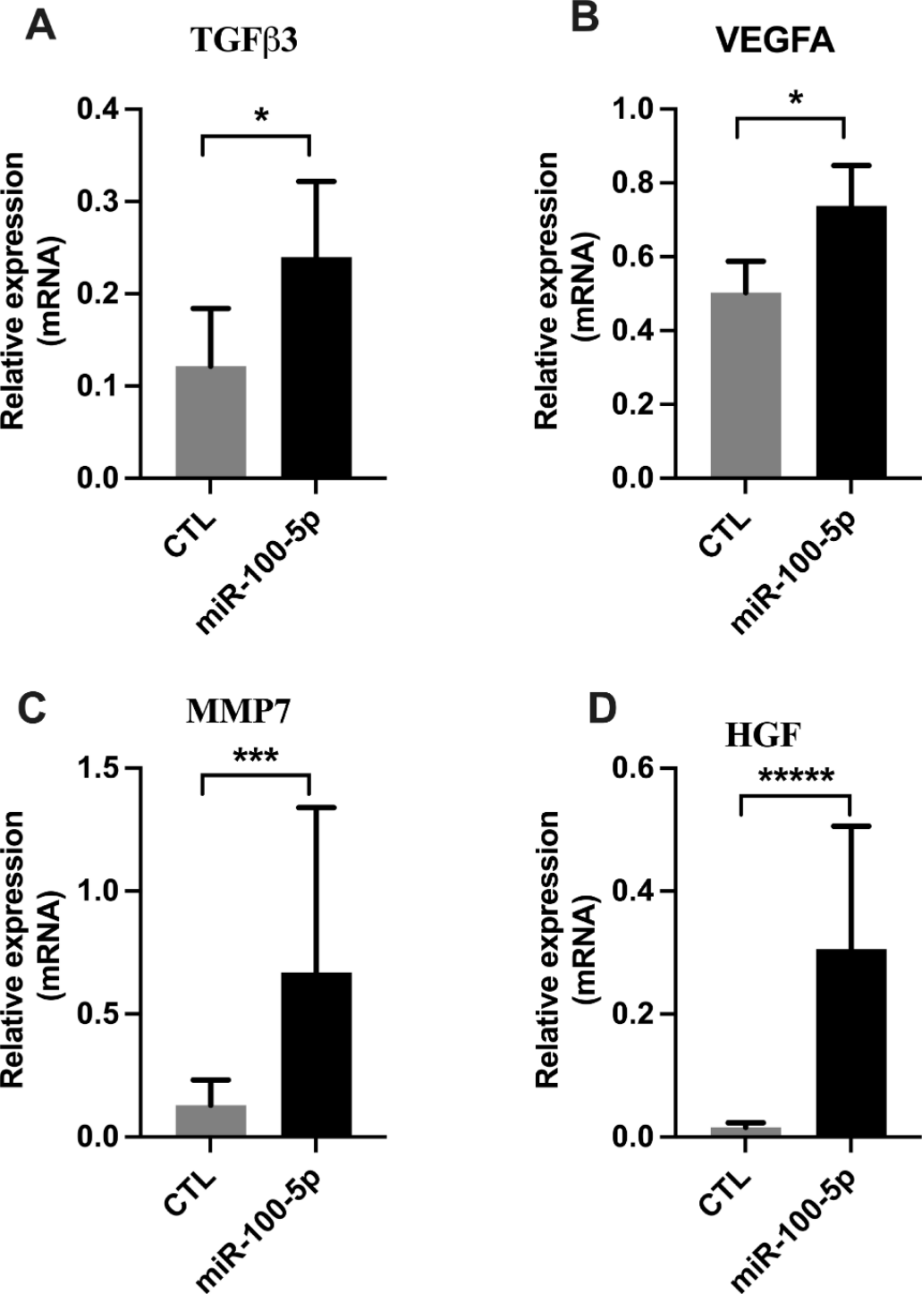
miR-100-5p upregulates regenerative markers in eSFs. Transfection of miR-100-5p in eSFs significantly increased the mRNA levels of regeneration markers (**a**) TGF-ß3, (**b**) VEGFA, (**c**) MMP7, and (**d**) HGF compared to controls. Each bar represents the data from four individual experiments, and each experiment was performed in triplicate. Data are shown as mean ± standard error (SEM) CTL vs. mimic (*p < 0.05), ***p < 0.001, **** *p* < 0.0001)

### MiR-100-5p induced regeneration of endometrial cells

To identify the role of miR-100-5p and miR-143-3p in tissue regeneration, we used eSFs for their regenerative potential. We found that transfection of eSFs with miR-100-5p significantly increased mRNA levels of TGFβ3, VEGFA, MMP7 and HGF (*p* < 0.05) as shown in Fig. 4. These results suggest that miR100-5p, which is highly expressed in EVs from h-BMDSCs, is likely to enhance the regenerative capacity of eSFs. We did not observe any effects on regeneration of endometrial cells by miR-143-3p.

**Fig. 5:**
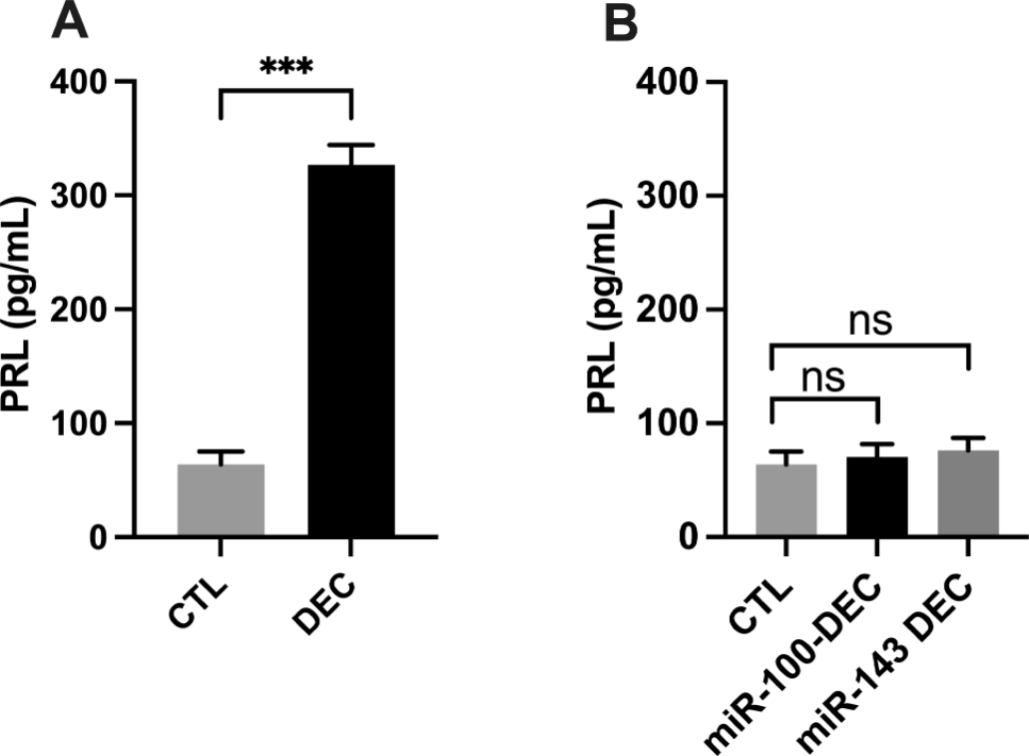
Inhibition of decidualization by miR-100-5p and miR-143-5p. eSFs with and without transfection of miR-100-5p and miR-143-5p were treated with cAMP + MPA. Prolactin was estimated in conditioned medium by ELISA. (**a**) Shows significant increase in PRL levels after 7 days in normal cells treated with cAMP + MPA compared to no treatment. (**b**) Shows no significant increase in PRL levels in cells transfected with miRNAs compared to cells without transfection. Each bar represents the data from two individual experiments, and each experiment was performed in duplicate. Data are shown as mean ± standard error (SEM) CTL vs. Decidualization (*** *p* < 0.001)

### miR-100-5p inhibited differentiation/decidualization of eSFs

To know the function of miR-100-5p and miR-143-3p in cell differentiation/decidualization, we used an *in-vitro* model of decidualization where eSFs were treated with medroxy- progesterone in combination with cAMP. After 7 days, the prolactin (PRL) levels were measured from the conditioned media using ELISA. Prolactin levels were found to be significantly greater in non-transfected cells than in cells transfected with miR-100-5p and miR-143-3p (Fig. 5a & b).

**Fig. 6:**
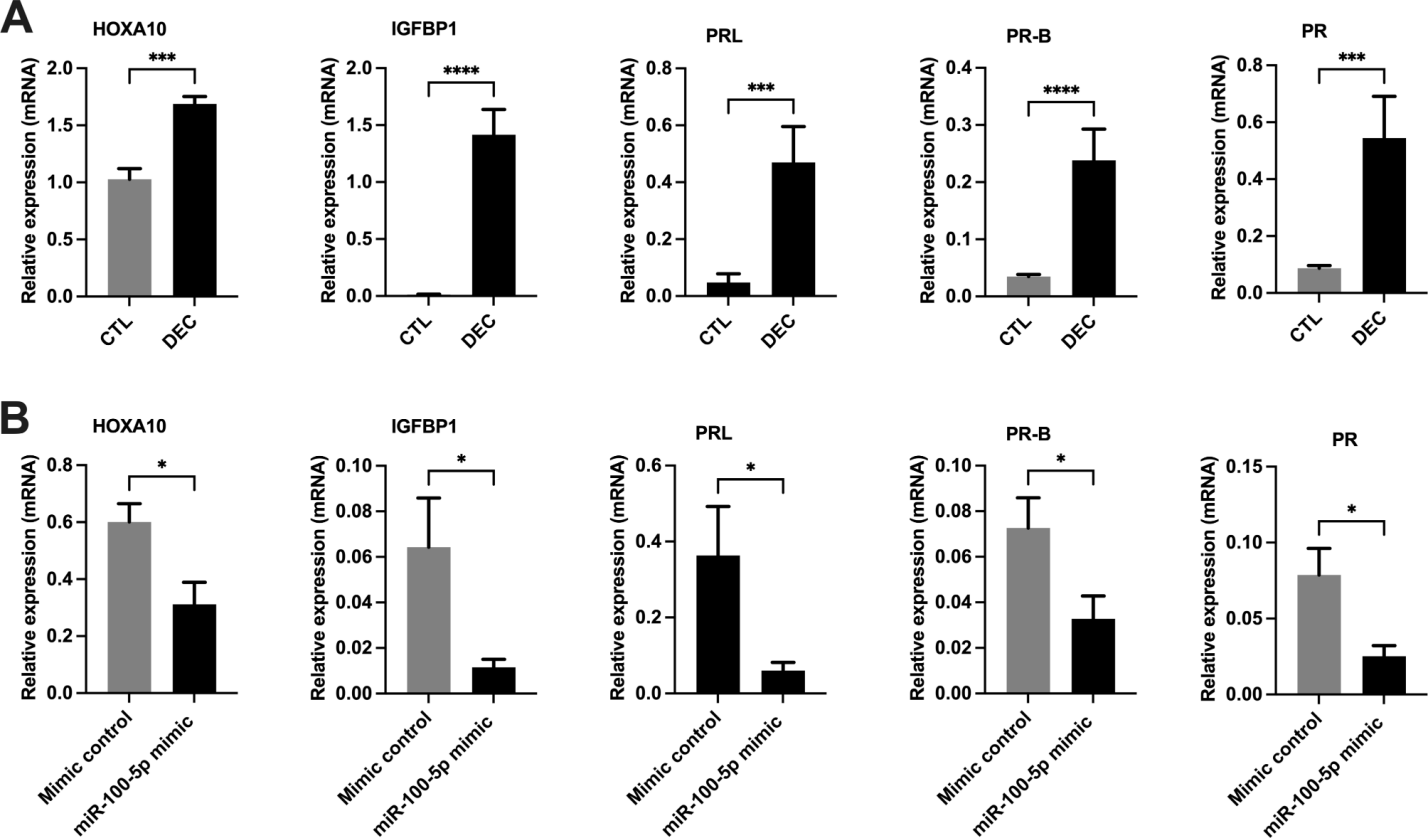
Effect of miR-100-5p on decidualization markers. mRNA levels of decidualization markers were determined by qRT-PCR. (**a**) Shows the significant upregulation of mRNA levels of decidualization markers HOXA10, IGFBP-1, PRL, PR-B, and PR in normal eSFs treated with cAMP + MPA for 7 days (*DEC*), compared to control group (*CTL*). (**b**) Shows that miRNA miR-100-5p significantly downregulates mRNA levels of decidualization markers HOXA10, IGFBP-1, PRL, PR-B and PR significantly in eSFs compared to non-transfected cells with the mimic control. Each bar represents the data from four individual experiments, and each experiment was performed in triplicate. Data are shown as mean ± standard error (SEM) CTL vs. DEC and mimic negative control vs. miR-100-5p transfection. (*p < 0.05), **p < 0.01, ***p < 0.001)

**Fig. 7:**
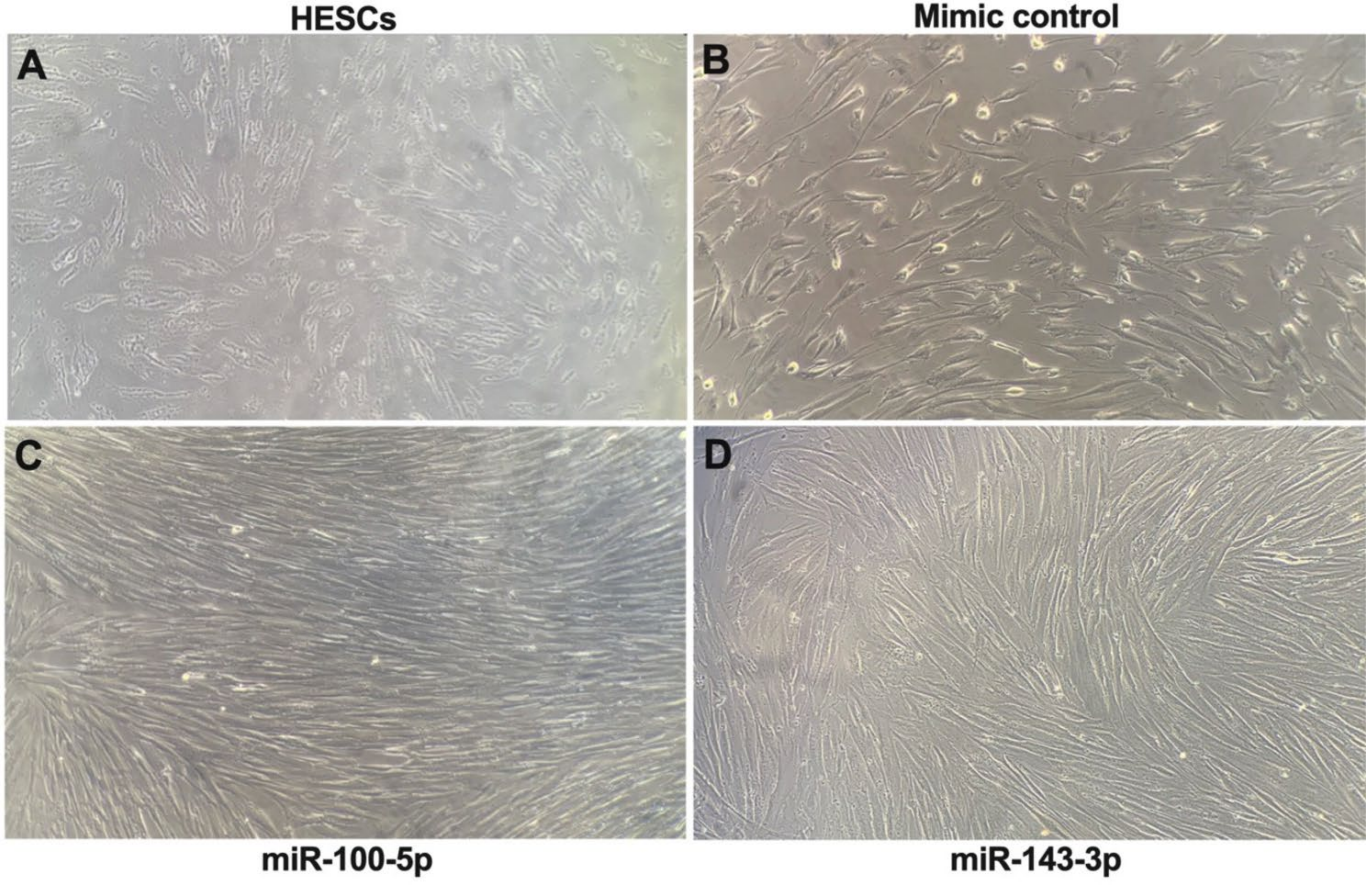
Inhibition of decidualization of eSFs in vitro by miR-100-5p and miR-143-3p. (**a**) Normal eSFs (HESCs), (**b**) eSFs transfected with miRNA mimic control. Cells gradually changed to typical decidual cells after 7 days of cAMP + MPA treatment. (**c** & **d**) eSFs transfected with miR-100-5p and miR-143-3p respectively. No typical or partial decidual changes were observed after 7 days treatment of cAMP + MPA

To determine the effect of miR-100-5p and miR-143-3p on decidualization, we induced decidualization in eSFs alone, eSFs transfected with miR-100-5p and miR-143-3p mimics, and the negative controls. Figure 6a shows a significant increase in mRNA levels of the decidualization markers HOXA10 (1.7-fold), IGFBP-1 (150-fold), PRL (7-fold), PR-B (5-fold), and PR (10-fold), in eSFs treated with cAMP + MPA. eSFs transfected with miR-100-5p mimic, significantly decreased mRNA levels of all decidualization markers (HOXA10 (2-fold), IGFBP-1 (6-fold), PRL (5-fold), PR-B (2.5-fold) and PR (8-fold) compared to controls (Fig. 6b). We did not observe any significant decrease in mRNA levels of these markers in eSFs transfected with miR-143-3p during induced decidualization, the morphological changes in cell structures were also recorded. eSFs transfected with the negative control became rounder with increased cell volume and ambiguous cell boundaries, which are typical features of decidual cells (Figs. 7b) compared to normal cells (Fig. 7a). These morphological changes were inhibited in cells transfected with either mir-100-5p or 143-3p as showed in Fig.  **7** c & d respectively. These data suggest that miR-100-5p, block the endometrial cell differentiation, allowing for continued proliferation and tissue repair.

## Discussion

In this study, we described the role of the microRNAs miR-21-5p, miR-100-5p, miR-143-3p which are carried and transported by EVs (specifically exosomes) from hBMDSCs in tissue regeneration and decidualization using a eSF cell line *in-vitro*. Our results revealed that these miRNAs, which are highly abundant in hBMDSCs-derived exosomes, induce stromal cell proliferation and tissue regeneration while inhibiting the decidual differentiation of hormone-mediated stromal cells in the endometrium. Tissue regeneration requires a pause in the normal differentiation process to allow cell proliferation and repair. Specific miRNAs from EVs derived from hBMDSCs can influence the endometrial regenerative capacity and therefore participate in regeneration.

MSCs are crucial in the maintenance of tissue homeostasis and restoration after injury. MSCs were first isolated from mouse bone marrow [29] and are known to differentiate into several non-hematopoietic cells of visceral mesodermal, endodermal and ectodermal tissues [6, 8]. However, humans have a limited capacity to regenerate, and the extent of this regenerative capacity varies among different adult tissues. Each tissue has an inherently different cellular turnover and repair potential [1]. hBMDSCs were thought to maintain tissue homeostasis by serving as stem cell reservoirs for tissue repair and regeneration. Additionally, MSCs are thought to drive the fate of endogenous tissue-resident progenitor cells and to participate in adult cell plasticity [30, 31]. However, recent evidence suggests, that the principal action of BMDSCs is likely due to bidirectional intercellular paracrine communication [7, 10]. Low stem cell engraftment in target tissues is insufficient to justify their often-dramatic effects, supporting the idea that the final effectors of regenerative processes are likely to be paracrine soluble factors released by the stem cells.

Analysis of miR-21-5p data especially IPA analysis suggest that it mediates tissue regeneration, including stress response and inhibition of senescence and fibrosis. MiR-21 has been linked to the regulation of skin damage and wound re-epithelization through regulating angiogenic and inflammatory pathways [32]. Exosomal miR-21 derived from M2.

macrophages was recently found to promote angiogenesis and wound healing, leading to a successful regeneration of skin functional microstructure *in-vivo* [33]. Similarly, MiR-21-loaded and internalized exosomes have been associated with spinal cord injury repair [34], axonal regeneration [35], osteogenesis [36], liver regeneration, and lung regeneration [37]. We identified several mRNAs regulated by miR-21 that are likely to contribute to the regeneration of embryonic and adult tissues (including the endometrium) by promoting cell cycle quiescence, cell differentiation, migration, and/or angiogenesis [38–42]. Some have been also implicated in the reversal of cellular senescence [43–45], the attenuation of fibrosis [45, 46] and the stress response [47, 48]. Specifically, BDKRB1, a bradykinin G-coupled receptor B1, was significantly upregulated in eSFs by miR-21. A recent study, using transcriptomic analyses, revealed that its overexpression may contribute to the BMDSC trans-differentiation into the endometrial stromal lineage in the endometrium [49], reinforcing the idea that this microRNA may be affecting resident progenitor cells [50]. Similarly, we found that ERRFI1 was significantly downregulated by miR-21 in eSFs, consistent with its suppressive effects on endometrial cell proliferation [51]. Finally, upregulation of UCP2 was found to be essential for the prevention of induced oxidative stress in eSFs [52]. By IPA, we noted that IL-17 signaling was significantly activated in eSFs treated with miR-21, indicating that this immunomodulatory and metabolic pathway plays an essential role in tissue homeostasis and repair [53]. IL-17 signaling promotes cell proliferation and coordinate, tissue repair and wound healing in several tissues following injury or inflammation [54–57]. IL-17 signaling is pleiotropic, and outcomes are influenced by several signals present in the same environment, potentially contributing to IL-17-driven homeostasis, diseases, or aging [53, 55]. Given that this effect is likely context-dependent modulation of cytokine production by stem cells (rather than constitutive IL-17 signaling) may be advantageous in tissue repair.

Interestingly, we found that hsa-miR-100-5p promoted gene expression of four crucial angiogenic and reparative factors related to endometrial regeneration (TGFβ3, VEGFA, MMP-7 and HGF), thus reinforcing the reparative potential of miRNA-derived exosomes on eSFs. HGF is a pleiotropic factor which can promote the motility, migration, and proliferation of a wide spectrum of cells. HGF has been implicated in endometrial remodeling and cell proliferation during both the menstrual cycle (under estrogen influence) and via auto/paracrine mechanisms [58, 59]. VEGFA is increased at the time of endometrial repair and regeneration, mostly during the endometrial proliferative phase, both in humans [60] and primates [61]. Additionally, progesterone withdrawal is necessary for this pre and post-menstrual expression. MMPs are differentially expressed during the menstrual cycle, and therefore play a significant role in normal endometrial remodeling via degradation of the extracellular matrix. MMP-7 specifically is required for the reepithelization of mucosal wounds [62] and is highly expressed during the endometrial proliferative phase, exerting functions associated with tissue regeneration and angiogenesis [63–65]. Isoform 3 of TGFβ is also highly expressed during the proliferative phase [66] and has recently been identified as a potential regenerative anti-scarring therapy for skin [67]. Moreover, in the luteal phase, progesterone strongly inhibits both MMP-7 [68, 69] and TGFβ3 [70], consistent with the concept that differentiation and regeneration are distinct cellular processes that cannot occur simultaneously. Taken together, our data suggest that BMDSC-derived miRNAs promote proliferation and tissue repair remodeling.

Further, our study revealed that hsa-100-5p miRNA prevented the differentiation/ decidualization of eSFs by downregulating the HOXA10, IGFBP-1, PRL, PR-B, PR and genes which are the drivers of decidualization. This is consistent with the need to prevent differentiation in early endometrial regeneration/ repair. In a recent cross-sectional study, live birth rate was negatively associated with circulating miR-100-5p in women prior to beginning in vitro fertilization [71]. Similarly, miR-100-5p was found to be significantly upregulated in both plasma and decidual tissues during early pregnancy of women with recurrent miscarriage compared to controls [72]. Repair processes in the endometrium require that decidual differentiation is blocked to allow for proliferation, repair and regeneration. Thus, both processes may be reciprocally regulated by the same miRNAs. We did not observe any significant changes in the decidualization process for miR-143-3p.

The successful approval of MSC-based therapies has set the stage for regenerative medicine to become a common treatment modality for several degenerative diseases related to aging or abnormal/absent tissue regeneration [73, 74]. In particular, hBMDSCs have emerged as the most commonly used therapeutic cells because of their pleiotropic effects, intrinsic ability to migrate and engraft into target tissues, their renewal, and multipotent differentiation potential. They also provide a strong paracrine support for several physiological or pathological conditions [75, 76].

Taken together, our results suggests that hBMDSCs have a powerful and multifaceted paracrine effect on endometrial homeostasis by shuttling specific miRNAs via exosomes. These exosomal microRNAs have the ability to enhance the proliferative/regenerative potential and simultaneously prevent terminal differentiation, thus allowing for tissue proliferation and repair. Limitations of direct MSC-based therapy are post-administration cell loss, safety, and variability in therapeutic response. MSC-derived EVs are attractive candidates for cell-free therapies in regenerative medicine as they have shown stability in the extracellular biofluids, excellent biocompatibility, and low toxicity and immunogenicity [77]. Moreover, EVs can cross the blood-brain barrier, allowing them to be a feature considered superior to the traditional MSC-based therapies.

## Conclusions

We identified the major microRNAs present in EVs secreted by hBMDSCs, and demonstrated that the miRNAs miR-21-5p, miR-100-5p, and miR-143-3p are involved in regulating the genes responsible for endometrial regeneration and decidualization. To determine whether the hBMDSCs-derived microRNAs can be used as therapeutic agents, *in-vivo* studies are needed to confirm our *in-vitro* studies. These studies will help in replace stem-cell-based cellular therapies with cell free treatments using biomolecules such as microRNAs, thus serving as new therapeutics for tissue regeneration and repair.

### Electronic supplementary material

Below is the link to the electronic supplementary material.

Supplementary Material 1

Supplementary Material 2

Supplementary Material 3

## Data Availability

Data available with Ramanaiah Mamillapalli and Hugh Taylor.

## Abbreviations

miRNAs: microRNAs
h-BMDSCs: human bone marrow-derived stem cells
EVs: extracellular vesicles
TEM: transmission electron microscopy
NTA: nanoparticle tracking analysis
eSFs: human endometrial stromal cells
MSCs: mesenchymal stem cells
qRT-PCR: quantitative real-time polymerase chain reaction
TGFβ3: transforming growth factor-beta3
VEGFA: vascular endothelial growth factor A
MMP7: matrix metalloproteinase-7
HGF: hepatocyte growth factor
HOXA10: homeobox A10
IGFBP-1: insulin-like growth factor-binding protein 1
PRL: prolactin
PR-B: progesterone-B
IPA: ingenuity pathway analysis
