# Supplementary figures and images for: Bone marrow mesenchymal stem cell-derived exosomes shuttle microRNAs to endometrial stromal fibroblasts that promote tissue proliferation /regeneration/ and inhibit differentiation
Source: Stem Cell Res Ther. 2024 May 1;15:129. doi: 10.1186/s13287-024-03716-1 (PMC11064399; doi:10.1186/s13287-024-03716-1)

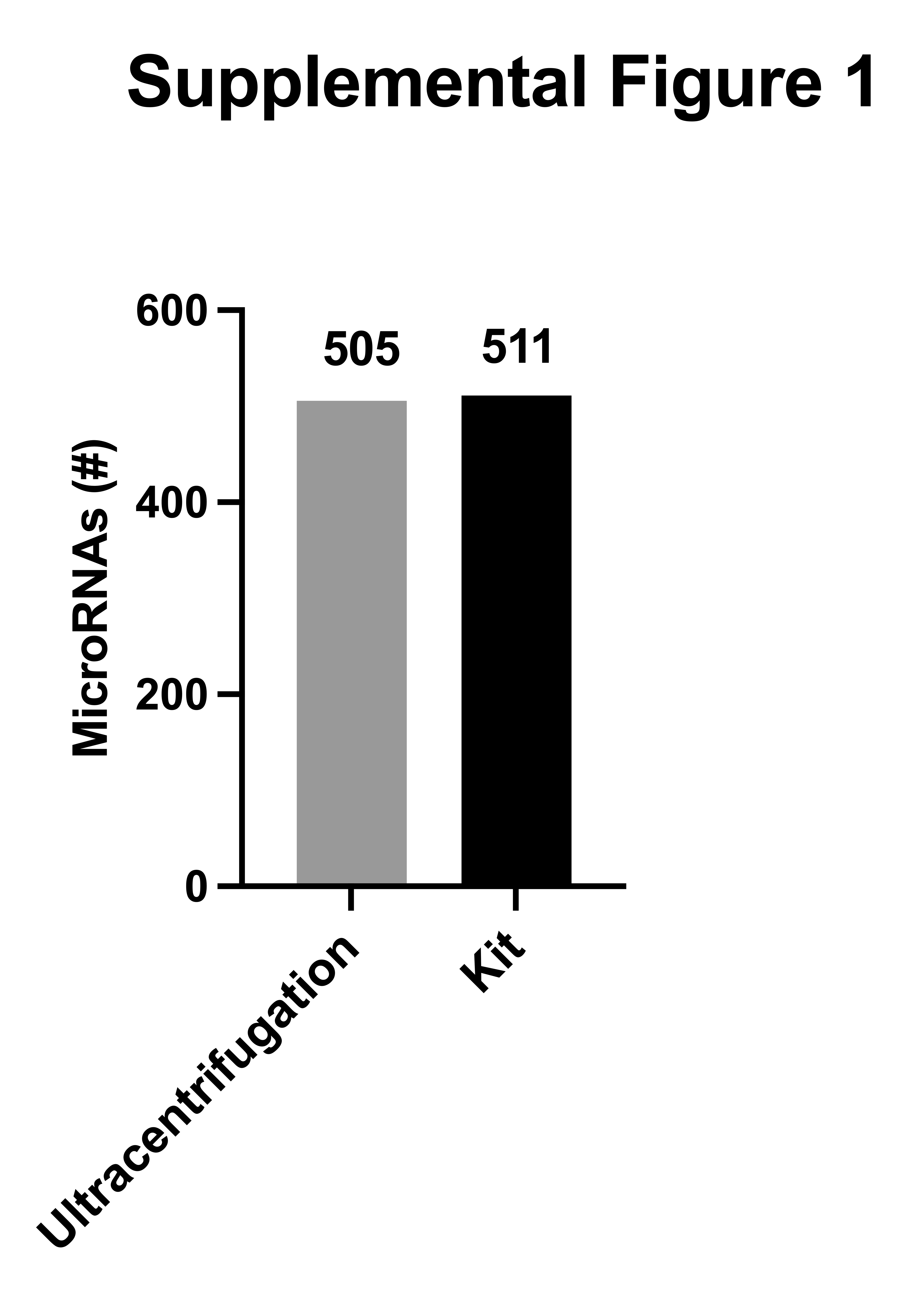

Supplement: Supplementary file 1 — Supplementary Material 1 [file 13287_2024_3716_MOESM1_ESM.tiff]

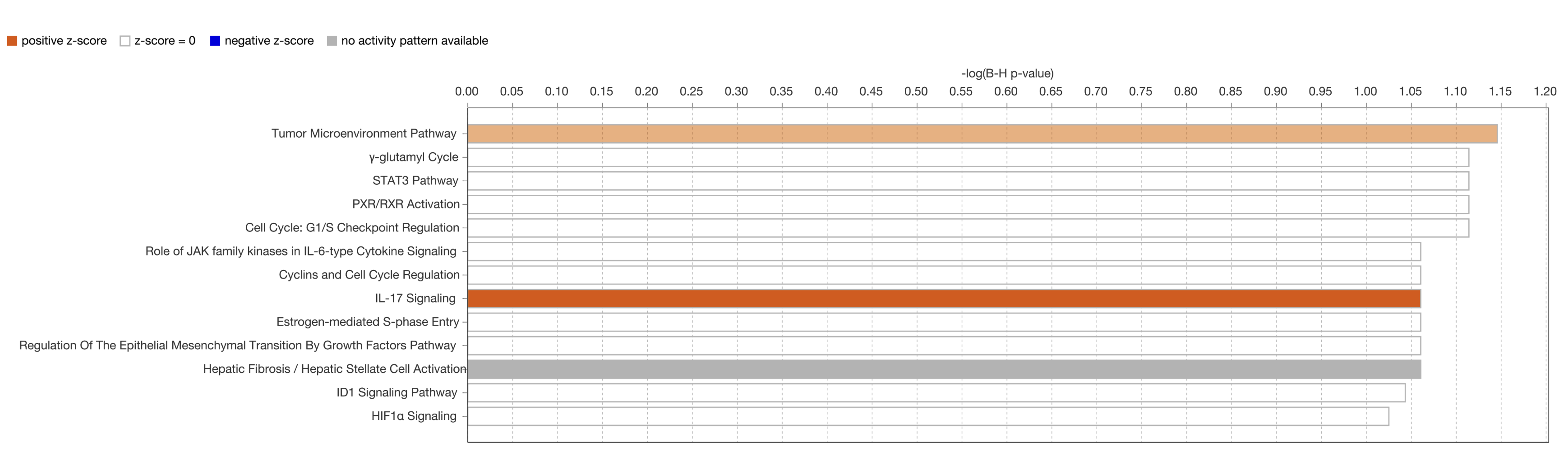

Supplement: Supplementary file 2 — Supplementary Material 2 [file 13287_2024_3716_MOESM2_ESM.tiff]

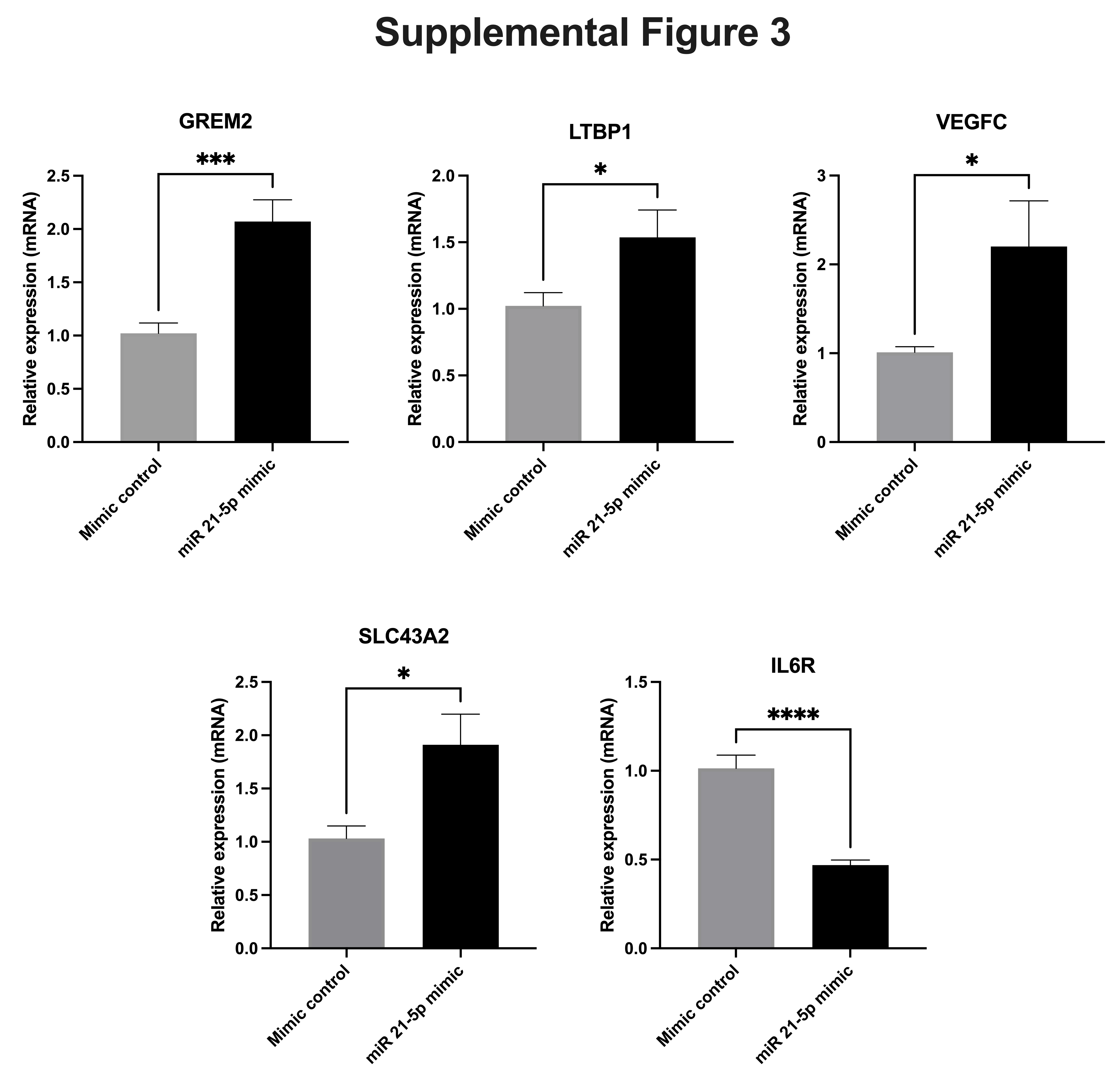

Supplement: Supplementary file 3 — Supplementary Material 3 [file 13287_2024_3716_MOESM3_ESM.tiff]
